# Clinical Outcomes, Success/Failure Patterns, and Complications of Microscrew-Assisted Rapid Palatal Expansion in Post-Pubertal Transverse Maxillary Deficiency: A Scoping Review

**DOI:** 10.3390/dj14050261

**Published:** 2026-05-01

**Authors:** Claudia Butrón-Téllez Girón, Juan Carlos Flores-Arriaga, Daniel Oliva-Buhaya, Alan Martínez-Zumarán, Amaury Pozos-Guillén, Arturo Garrocho-Rangel

**Affiliations:** 1 Dental Sciences Master Program, Faculty of Dentistry, University of San Luis Potosí, San Luis Potosí 78290, Mexico; claudia.butron@uaslp.mx (C.B.-T.G.); carlos.flores@uaslp.mx (J.C.F.-A.); 2 Orthodontics Postgraduate Program, Faculty of Dentistry, University of San Luis Potosí, San Luis Potosí 78290, Mexico; luisoliva24@hotmail.com (D.O.-B.); alanzuma@uaslp.mx (A.M.-Z.); 3 Pediatric Dentistry Postgraduate Program, Faculty of Dentistry, University of San Luis Potosí, San Luis Potosí 78290, Mexico; apozos@uaslp.mx

**Keywords:** MARPE, palatal expansion technique, orthodontic anchorage procedures, treatment failure, risk factors, adolescents/young adults, side effects and adverse reactions

## Abstract

**Background/Objectives**: A non-surgical orthodontic treatment strategy for transverse maxillary deficiencies, especially in late adolescents and young adults, is microscrew-assisted rapid palatal expansion (MARPE). The literature indicates several concerns regarding its long-term efficacy and potential complications. Recent studies have provided valuable insights into the MARPE technique, particularly focusing on its efficacy, potential complications, and treatment failures. The present scoping review aims to synthesize and critically appraise clinical evidence on MARPE in post-pubertal patients, with a specific focus on treatment outcomes, mechanisms of failure, and local and systemic adverse effects to inform risk–benefit assessment and clinical decision-making. **Methods**: A systematic search was conducted across four electronic databases (PubMed, EMBASE, Scopus, and Cochrane Library) to identify English-language clinical trials, observational studies, and systematic reviews published between January 2015 and December 2025. The search strategy employed controlled vocabulary (MeSH terms) and Boolean operators targeting MARPE, treatment failure, and adverse effects in patients aged ≥ 16 years. After title/abstract screening and full-text assessment using predetermined inclusion criteria, 15 studies (3 systematic reviews with meta-analysis, 2 umbrella reviews, 4 systematic/scoping reviews, 2 randomized controlled trials, and 4 observational studies) were selected for qualitative synthesis. **Results**: Fifteen studies were finally included, which demonstrated significant heterogeneity in methodological design, sample characteristics, outcome measurement protocols, and MARPE device specifications. Mean success rates of 92.5% for maxillary transverse expansion were reported, with mean expansion duration ranging between 20 and 126 days. Key adverse effects comprised dentoalveolar tipping (buccal inclination of maxillary molars and premolars), periodontal complications (buccal bone resorption of 0.6–0.9 mm, gingival recession, papilla recession in 18% of cases), root resorption, miniscrew loosening, midpalatine/circummaxillary sutures, and potential but minimally documented intracranial effects. **Conclusions:** MARPE appears to be a valid non-surgical option for selected post-pubertal patients, but its success depends on careful case selection and monitoring for dentoalveolar, periodontal, sutural, and rare intracranial adverse effects.

## 1. Introduction

Fewer than 10% of individuals present maxillary transverse deficiency in their permanent teeth, compared to 8% in primary teeth and 23% in mixed dentition [1]. Rapid Palatal Expansion (RPE) is a well-known orthodontic technique that involves widening the upper jaw to correct maxillary transverse deficiencies. Traditional RPE methods rely on dental anchorage, which can induce some undesirable effects [2,3]. These side effects have promoted the development of alternative methods that provide more skeletal anchorage, reducing the risk of dental complications. One such innovation is Micro-Implant Assisted Rapid Palatal Expansion (MARPE), which uses temporary anchorage devices (TADs), commonly termed miniscrews or micro-implants, to enhance the skeletal outcomes of palatal expansion without any type of surgical intervention [4]. MARPE is a rigid orthodontic appliance with four miniscrews inserted into the para-midsagittal palatal region. The appliance delivers the expansion force directly to the basal bone and allows a true palatal disjunction, thus maximizing the skeletal effect in post-pubertal patients [5,6,7]. MARPE’s exerted force is applied mainly in the superior orbital fissure, oval foramen, round foramen, spinous foramen, and optic foramen [8]. The average duration of expansion with MARPE varies between 20 and 126 days, with reported success rates averaging 92.5% [9,10].

Maxillary transverse deficiencies are a common orthodontic condition, often leading to functional and esthetic problems, including crossbites and dental crowding [11]. The traditional RPE approach, while often effective, is not without limitations—especially in adults, where the mid-palatal suture is more resilient to expansion [12]. The advent of MARPE offers a promising solution by directly transferring the expansion forces to the skeletal structure, thus minimizing dental movements and enhancing skeletal outcomes. This appliance is considered a simple non-surgical orthodontic technique with minimal discomfort to the patient and lower costs [3].

Temporary anchorage devices (TADs) encompass a heterogeneous group of miniscrew and miniplate systems designed to provide skeletal anchorage for orthodontic tooth movement and orthopedic corrections, thereby reducing unwanted reciprocal dental effects [1,6,7]. Within this spectrum, palatal miniscrews have been increasingly used to anchor maxillary expansion appliances, giving rise to hybrid tooth- and bone-borne devices and fully bone-borne expanders [10,11]. Microscrew-assisted rapid palatal expansion (MARPE) refers to a class of expanders in which one or more jackscrews are rigidly connected to palatal miniscrews placed in the paramedian or midpalatal region, allowing transverse forces to be transmitted primarily to the basal bone rather than to the dentition [1,4,10]. In late adolescents and adults with transverse maxillary deficiency, MARPE has been proposed as a non-surgical alternative to conventional tooth-borne rapid palatal expansion and surgically assisted rapid maxillary expansion, maximizing skeletal expansion while minimizing dentoalveolar side effects [3,12].

The clinical purpose of MARPE is to achieve maxillary expansion without the need for surgery. Therefore, the device can maximize skeletal expansion by directly transferring the expansion force to the palatal surface of the maxilla [7]. However, this device can produce dentoalveolar effects and a negative impact on periodontal health [5,11]. Moreover, as patients grow older, their palatal structures become less flexible, and the chances of successful expansion will decrease as the number of suture fusion cases rises [13]. To date, research on MARPE has highlighted several positive skeletal and functional effects—particularly in adolescent and young adult patients—including maxilla widening in cases of posterior crossbites and tooth/arch size discrepancies, or increases in the upper airway space and airflow (e.g., transverse width of the nasal cavity), with minimal undesirable dentoalveolar effects [7,13,14]. Despite its proven clinical effectiveness, the technique is not without controversy, particularly regarding its potential adverse effects. These effects include dentoalveolar tipping rather than bone expansion, periodontal effects (e.g., gingival recession and dehiscence), root resorption in involved supportive teeth, risk of implant failure, and possible long-term consequences on the craniofacial complex [15,16,17]. Therefore, challenges such as implant stability, patient discomfort, and the technique’s complexity continue to be areas of concern [9,18]. Furthermore, there is ongoing debate regarding the longstanding stability of the results attained with MARPE and the potential for relapse over time [19].

The recently proposed skeletal transverse dimension (STD) framework by Watted et al. [20] underscores that accurate three-dimensional differentiation between skeletal, dentoalveolar, functional, and combined transverse discrepancies is a prerequisite for rational selection of maxillary expansion strategies in adolescents and adults. Within this diagnostic context, MARPE should be considered only after a comprehensive STD-oriented assessment has confirmed a predominantly skeletal maxillary deficiency and has delineated the limits of dentoalveolar compensation, thereby reducing the risk of inappropriate case selection that could lead to excessive buccal tipping, cortical dehiscence, or gingival recession. Integrating STD-based diagnostics, through study models, clinical evaluation, and CBCT, into routine work-up enables clinicians to distinguish relative from absolute transverse discrepancies, to identify when mandibular morphology or functional shifts contribute substantially to the transverse problem, and to determine when surgically assisted expansion (e.g., SARME/SARPE) or segmental osteotomy may offer a more predictable and stable correction than MARPE in complex adult cases.

This scoping review synthesizes and critically appraises contemporary clinical evidence on the microscrew-assisted rapid palatal expansion (MARPE) technique in post-pubertal patients, with particular emphasis on treatment outcomes, failure mechanisms, and local and systemic adverse effects. The primary objectives are to (1) provide a structured synthesis of the current state of knowledge regarding MARPE outcomes and complications; (2) identify and characterize documented causes of treatment failure; (3) systematically describe potential adverse effects, including dentoalveolar, periodontal, and intracranial consequences; (4) identify substantial gaps in the existing literature; and (5) offer recommendations for future, methodologically robust clinical research. In addition to treatment success/failure and adverse events, the review also sought to chart evidence on (1) periodontal effects, (2) sutural maturation and resistance structures, (3) biomechanical determinants of treatment efficacy, and (4) intracranial effects, as these domains are closely linked to the predictability, complication patterns, and long-term clinical stability associated with MARPE.

By integrating this evidence, the review aims to support clinicians in making evidence-informed decisions regarding case selection, diagnostic work-up, treatment planning, and clinical monitoring when using MARPE to manage transverse maxillary deficiencies in late adolescents and young adults.

## 2. Methods

### 2.1. Design

The present scoping review was created using the suggested methods by Arksey and O’Malley [21], Levac et al. [22], and the Preferred Reporting Items for Systematic Reviews and Meta-analysis guidelines for Scoping Reviews (PRISMA-ScR) [23]. This type of literature review maps and synthesizes the pertinent investigations on a wide variety of topics related to the essential ideas supporting a clinical subject of interest [22,24]. Globally, a scoping review encompasses the following stages: (i) design of a specific research question, (ii) search and identification of pertinent studies, (iii) selection of best studies, (iv) collection and charting of relevant data, and (v) organization, summarization, and reporting of the main results and findings.

This study did not aim to generate pooled estimates but, instead, to map and synthesize all clinically relevant evidence on MARPE failure mechanisms and adverse effects in post-pubertal patients over the last decade. Within this a priori framework, the strict eligibility criteria, clinical human studies, MARPE-specific interventions (as opposed to mixed RPE designs), a minimum post-pubertal age threshold, and explicit reporting of failure causes and adverse outcomes substantially reduced the pool of studies that were methodologically comparable and directly informative for the review question. The present scoping was registered on the Open Science Framework platform under the Project DOI 10.17605/OSF.IO/9U8DM.

### 2.2. Question Research

The search strategy was carried out for the most appropriate studies published in the previous 10 years on the MARPE orthodontic technique, its potential causes of treatment failure, and local and systemic adverse effects in patients older than 16 years of age. The search process was from 10 December 2024 to 18 December 2025. The demarcated PICO (“**P**opulation”-”**I**ntervention”-”**C**omparator”-”**O**utcomes”) question was: “In late adolescents and young adults with transverse maxillary deficiency (P), treatment with microscrew-assisted rapid palatal expansion (MARPE) (I), compared with conventional tooth-borne rapid palatal expansion and/or surgically assisted rapid maxillary expansion (C), is associated with which treatment outcomes, failure mechanisms, and local or systemic adverse effects (O)”.

In this scoping review, the primary outcome of interest was the failure rate of MARPE, defined as incomplete or absent midpalatal and/or circummaxillary suture separation, or clinically unsuccessful maxillary expansion, leading to modification, supplementation, or discontinuation of the planned MARPE protocol. Secondary outcomes comprised the spectrum and frequency of reported adverse effects, including (i) dentoalveolar changes such as buccal inclination of anchor teeth and unwanted diastema characteristics; (ii) periodontal complications, including buccal bone loss, cortical dehiscence, and gingival or papillary recession; (iii) skeletal and sutural effects, such as asymmetric expansion or unfavorable alterations in resistance structures; and (iv) systemic or neuro-ophthalmologic events potentially associated with changes in intracranial pressure or cranial base biomechanics.

### 2.3. Eligibility Criteria

The eligibility criteria were defined a priori according to the PICO framework. Studies were included if they (1) involved human participants aged 16 years or older with transverse maxillary deficiency; (2) evaluated microscrew-assisted rapid palatal expansion (MARPE) or closely related miniscrew-assisted skeletal expanders as the main intervention; (3) reported at least one of the predefined outcomes, namely MARPE failure (incomplete or absent midpalatal and/or circummaxillary suture separation or clinically unsuccessful expansion) and/or adverse effects (dentoalveolar, periodontal, skeletal/sutural, or systemic/neuro-ophthalmologic); (4) were designed as randomized or non-randomized clinical trials, observational studies (cohort, case–control, or cross-sectional), or systematic, umbrella, or scoping reviews; and (5) were full-text articles published in peer-reviewed journals in English between 1 January 2015, and 31 December 2025. Studies were excluded if they were (1) case reports or very small case series without systematic outcome assessment; (2) conducted in growing patients younger than 16 years; (3) non-dental, in vitro, animal, or purely biomechanical modeling studies without clinical MARPE data; (4) studies in which MARPE was not the primary intervention or could not be analytically distinguished from conventional RPE or surgically assisted expansion; or (5) opinion papers, narrative expert reviews without systematic methods, letters to the editor, conference abstracts, theses, dissertations, grey literature, or non-English publications.

### 2.4. Study Screening Selection

Comprehensive electronic searches were conducted to identify potentially relevant citations across four databases—PubMed (via MEDLINE), EMBASE (via Ovid), Scopus, and the Cochrane Library—complemented by hand searching of reference lists from included studies. Studies published exclusively in English between 1 January 2015 and 31 December 2025 were considered eligible; grey literature was not consulted.

Diverse combinations of related keywords (and synonyms), free terms, MeSH phrases, and Boolean operators were used to create an electronic search strategy. The focal key search terms were “*microscrew-assisted rapid palatal expansion*,” “*MARPE technique*,” “*treatment failure*,” “*adverse effects*,” “*miniscrew-assisted rapid palatal expansion,* “*microimplant-assisted*,” “*MSE*,” “*skeletal expander*,” “*maxillary skeletal expander*,” “*complications*,” “*side effects*,” “*failure rate*,” “*non-surgical expansion*,” “*stability*,” “*relapse*,” “*periodontal*,” “*root resorption*,” “*nasal floor*,” “*pterygopalatine*,” *and* “*zygomaticomaxillary buttress”*.

For each database, tailored search strategies combining controlled vocabulary terms (e.g., MeSH/Emtree), free-text keywords, and Boolean operators were developed based on the PICO framework and the main concepts of MARPE, treatment failure, and adverse effects (according to the PRISMA ScR Compliance and Transparency Documentation). The complete search algorithms for all databases are provided in the Supplementary Table S1.

PubMed via MEDLINE: Full algorithm incorporating MeSH descriptors (e.g., “Palatal Expansion Technique” [MeSH], “Orthodontic Anchorage Procedures” [MeSH], “Treatment Failure” [MeSH], “Drug-Related Side Effects and Adverse Reactions” [MeSH], “Risk Factors” [MeSH], “Adolescent” [MeSH], “Young Adult” [MeSH]) combined with free-text All Fields searches and proximity operators.

EMBASE via Ovid: Complete Emtree controlled vocabulary search (exp palate expansion/, exp orthodontic anchorage/, exp treatment failure/, exp adverse drug reaction/, exp complication/, exp risk factor/, exp periodontal disease/, exp tooth resorption/) combined with free-text keyword searches and proximity operators (adj3, adj2).

Scopus: Title-Abstract-Keywords (TITLE-ABS-KEY) compound search incorporating all intervention, outcome, and population terms with appropriate logical operators and filters for publication date (2015–2025), language (English), and document type (articles and reviews only; excluding case reports, letters, editorials, notes).

Cochrane Library: Advanced Search Builder strategy employing both MeSH descriptors (exploded to all trees) and free-text ti, ab, kw (title, abstract, keyword) searches, with date restrictions and publication type filters specific to Cochrane’s structure.

Two precalibrated writers (DOB and CBT) carried out, independently and in parallel, the search process of titles and abstracts, following the predetermined inclusion and exclusion criteria. The reviewers were blinded to each other’s decisions during this initial phase. Cohen’s kappa coefficient was used to determine the levels of agreement between and within these writers. With consultation from the other four authors (JFA, APG, AMZ, and AG), any disagreement was settled by consensus and discussion. Every discovered record was immediately entered into an EndNote worksheet that had been previously created and tested; all authors then carefully reviewed the worksheet to look for any mistakes or discrepancies across the dataset. Duplicated studies were eliminated. Then, the dataset was imported into the Covidence^®^ platform, including the location of possible cross-referenced publications and the identification of citations. Full-text papers that might be relevant were retrieved and carefully examined. Two authors (AP and AG) created the final list of chosen studies for the scoping review; once more, disagreements were resolved by discussion and agreement with the other authors (DOB, JFA, AMZ, and CBT).

### 2.5. Risk of Bias Assessment

For the present scoping review, the risk of bias (methodological quality) of the included studies was planned to be assessed using design-specific, internationally recommended tools, applied independently by at least two calibrated reviewers with consensus resolution for any disagreement. For randomized controlled trials, the Cochrane RoB 2 tool was selected, as it is the current standard for evaluating bias across domains such as the randomization process, deviations from intended interventions, missing outcome data, outcome measurement, and selective reporting. For non-randomized intervention studies (e.g., prospective or retrospective cohorts, controlled clinical trials without randomization), the ROBINS-I tool was considered the most appropriate, given its structured assessment of bias due to confounding, selection, classification of interventions, deviations from intended interventions, missing data, outcome measurement, and selection of reported results. For purely observational analytical designs such as cohort and case–control studies, validated instruments derived from the Newcastle–Ottawa Scale (NOS) and its recent adaptations for observational research were prioritized, while for cross-sectional designs, design-specific NOS-based tools were favored to better capture biases related to sampling, measurement, and confounding. The overall results of these evaluations are shown in Table 1.

### 2.6. Data Charting and Synthesis of Results

Considering the objectives of the review and the research question, a data extraction process was performed to produce a narrative/descriptive summary of the findings. First, the author and year, country, study methodological design, intervention(s), results, risk-of-bias assessment, and key findings/conclusions were noted for each included publication. The target population, sample size, and follow-up period were also stated concerning clinical trials and observational/longitudinal studies. Three authors individually gathered each item (CBT, DOB, and JFA). Disagreements during the extraction process were resolved by consulting the other three writers (AMZ, AP, and AG).

## 3. Results

**Figure 1:**
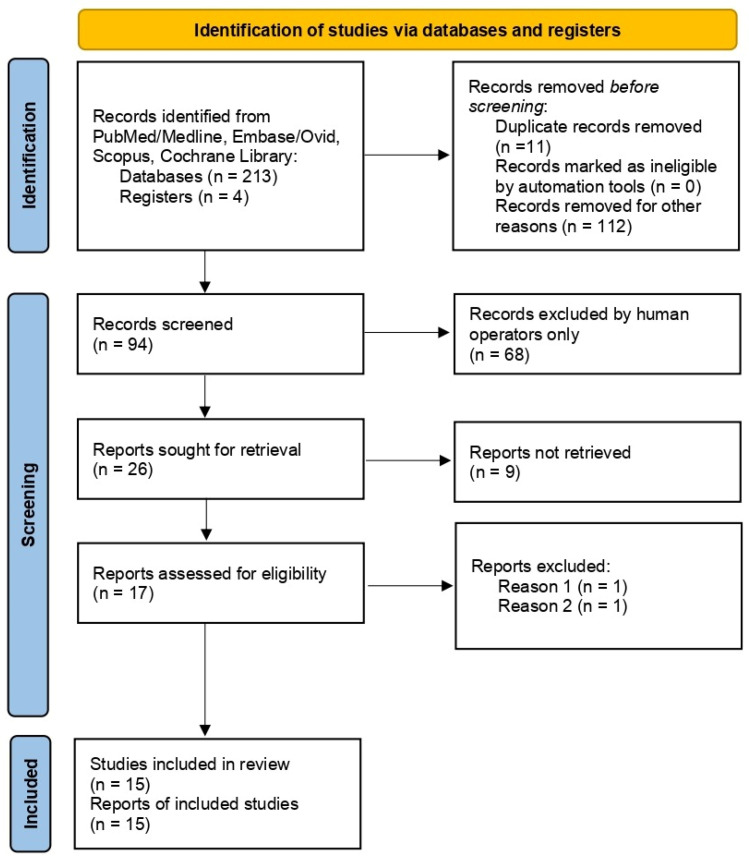
PRISMA ScR 2020 flow diagram of the selection process [23].

A total of 217 titles/abstracts/keywords were retrieved in the initial search (86 articles from PubMed, 70 from EMBASE, and 61 from Scopus and Cochrane Library). After removing duplicates and unrelated articles, 154 records were subsequently screened. This step resulted in 94 articles, of which 26 articles were selected, and 9 were omitted. Thus, 17 full-text articles were comprehensively evaluated and, finally, 15 were included for the present review (Figure 1). Significant heterogeneity was found among the included studies concerning methodological design, objectives, patient inclusion criteria, and sampling technique, among other characteristics (Table 1).

Of the fifteen studies selected, six were systematic/scoping reviews (three of them with meta-analysis) [9,18,25,27,28,29,30]; two were umbrella reviews [3,26]; two were randomized controlled clinical trials with two parallel arms [17,31]; and four were observational studies [7,32,33,34]. Most of these studies were performed in Asian, South American, and European countries. In accordance with the revised objectives, the Results and Discussion sections are presented not only in terms of MARPE success, failure, and adverse effects, but also by mapping conceptually related domains that may influence these outcomes. Specifically, the included studies reported data on periodontal responses, sutural maturation and resistance structures, biomechanical determinants of treatment efficacy, and, in an additional subset, potential intracranial effects, which are summarized in separate subsections to illustrate how these factors may modulate clinical predictability and risk profiles in late adolescent and young adult patients undergoing MARPE.

## 4. Discussion

According to the retrieved evidence, the probability of MARPE failure increases due to the fusion of midpalatal and other craniofacial sutures during the second and third decades of life [35,36]. Previous works have reported that the midpalatal suture (MPS) starts to fuse after 25 years and that, at this age, the suture is radiographically visible only in half of the population [5,37]. On the other hand, certain research has demonstrated that there is no absolute association between age and the moment of palatal suture fusion; for example, it has been shown that subjects between the ages of 32 and 71 may still not have fusion [27,28]. Additionally, computed tomography and histology have demonstrated that age is unreliable for palatal suture fusion [38,39].

Conventional RPE (e.g., Hyrax or Haas appliances) and other rapid palatal expansion methods are effective in treating transverse skeletal maxillary disharmonies in pre-teenage patients. Nevertheless, the technique rarely works well in adult patients because the MPS starts to fuse and stiffen. Additionally, various unfavorable outcomes associated with this approach have been documented, such as pain, tissue irritation and ulcerations, gingival retraction, root resorption, vestibular tooth’s crown inclination, alveolar bone dehiscence, decreased width and height of buccal and lingual bone, limited or unsuccessful skeletal expansion, unstable outcomes, and post-treatment relapse [18,27,33]. Instead, SARME, or Surgically Assisted Rapid Maxillary Expansion, is considered a straightforward and well-researched, albeit more invasive technique [18]. However, this strategy has also demonstrated the hazards that come with surgery, increased expenses, and a range of concerns, including asymmetry, epistaxis, postoperative pain, periodontal issues, and improper expansion [40].

A more clinically oriented interpretation of the present findings can be achieved by organizing the heterogeneous causes of MARPE failure and adverse effects into four interrelated domains: patient-related, appliance-related, process-related, and structure-related factors [31,41,42]. Patient-related determinants include chronological age and, more importantly, midpalatal suture maturation stage, baseline periodontal status and gingival biotype, and the condition of the nasal floor mucosa, all of which modulate individual susceptibility to incomplete suture opening, cortical dehiscence, and gingival recession under transverse loading. Appliance-related factors encompass miniscrew characteristics (length and diameter), the use of monocortical versus bicortical anchorage, and the three-dimensional position of the miniscrews and expansion screw body, which influence force transmission, primary stability, and the risk of root proximity or soft-tissue irritation. Process-related variables include the activation protocol (rate and duration of expansion), retention strategy (length of consolidation and post-expansion stabilization), and oral hygiene maintenance around miniscrews and appliance components, which together affect the balance between skeletal versus dentoalveolar responses and the incidence of peri-implant inflammation or soft-tissue complications. Finally, structure-related factors comprise the intrinsic resistance of craniofacial buttresses (zygomaticomaxillary buttress, crista zygomaticoalveolaris), the pterygomaxillary and other circummaxillary sutures, and the thickness and density of palatal cortical bone; these anatomic constraints largely determine the upper limit of non-surgical expansion and help identify patients in whom MARPE is likely to fail or must be supplemented or replaced by surgically assisted expansion techniques.

Possible adverse effects during rapid maxillary expansion when MARPE is used in adolescents and young adults with upper transverse deficiency will be described in detail and discussed below:

### 4.1. Periodontal Effects

MARPE can provide a suitable method for achieving orthopedic expansion of the maxillary basal bone, as well as having an impact on the circummaxillary sutures. Periodontal effects are usually mild in magnitude, and literature reports are still inconclusive [33]. As a result of the excessive stress applied by MARPE, the periodontal ligament experiences areas of compression, which can affect the supporting teeth’s periodontal health, including the reduction in both the alveolar bone crest (or apical shift, especially in the upper first premolar and first molar) and the thickness of the buccal bone (by 0.6–0.9 mm) [25,41,43,44,45]. According to Lim et al., the augmented interdigitation of the MPS with age might cause greater detrimental periodontal changes after maxillary expansion. Therefore, clinicians should carefully consider whether reducing buccal bone thickness has a negative consequence. According to Lim et al. [5], patients with thin buccal alveolar bone in the first premolar area and a low alveolar crest before expansion have a higher risk of alveolar dehiscence. Bone-borne SARPE may be a preferable option for patients who require severe maxillary transverse correction due to prior poor periodontium [5]. However, if it does occur, the periodontal harm may diminish with time. It has been shown that there is a physiologic remodeling process that can persist even after the appliance is removed. According to Ngan et al. [32], the decrease in buccal bone thickness can be recovered after 3 months, 6 months, or even 2 years after the MARPE treatment has finished. Therefore, the need for monitoring the patients over an extended period should be emphasized [5,41].

Buccal bone dehiscence, gingival recession, and significant changes in the dental crown height have also been reported immediately after maxillary expansion [5,25,33]. Chen et al. [33] stated that in 18% of subjects treated with MARPE treatment, the resulting diastema between the upper central incisors caused a mild gingival papilla recession. This kind of recession typically results in the appearance of a visually unappealing “black triangle,” which is challenging to repair even with periodontal regeneration procedures [28]. Concerning this, Brunetto et al. [45] mention that these periodontal damages are significantly less frequently observed in MARPE, regarding the already mentioned alternative palatal expansion techniques.

Contradictorily, other authors have stated that periodontal tissues remain healthy and firm during and after MARPE treatment [10,27]. For instance, in a study by Choi et al. [10], 31 subjects (19 and 35 years old) were treated with MARPE. The researchers did not find any significant dentoalveolar or periodontal side effects (e.g., moderate to severe pain, gingival damage, or bone dehiscence).

### 4.2. Dentoalveolar and Palate Adverse Effects

According to Marín et al. [46], first molars and alveolar structures have a higher risk of experiencing an undesirable significant buccal inclination during the use of MARPE. Lim et al. [5] reported a post-treatment inclination in the first molar’s crown by 3.91° [5,28,33].

A primary drawback of MARPE includes the microimplants’ invasiveness and the challenge of maintaining adequate palatal hygiene [1]. Transitional soft-tissue inflammation in the palate and the increased risk of local infection surrounding the MARPE miniscrews have been recorded during expansion treatment. After the allotted period for expansion treatment has passed, removing the appliance tends to reduce local gingival inflammation. Consequently, there is no sign of an interruption in treatment [27].

The length of MARPE miniscrews (1.8 mm × 9 mm or 1.5–1.8 mm × 11 mm) is one of the factors during the design of the appliance that can affect orthodontic outcomes and the occurrence of side effects. Longer miniscrews, which are inserted into the bicortical bone (palatal and nasal cortical bone), have been suggested to have superior orthopedic effects. However, this type of miniscrew can harm the nasal floor mucosa or produce discomfort to the patient; thus, the appliance should be closely observed [10].

### 4.3. Suture Maturation, Resistance Structures, and Biomechanical Determinants of Treatment Efficacy

From an anatomical and biological perspective, MPS maturation represents a critical determinant of treatment efficacy, with progressive ossification and interdigitation occurring throughout adolescence and early adulthood. Cone-beam computed tomography (CBCT)-based evidence has established that suture maturation follows distinct morphological stages, with stages D and E characterized by advanced fusion and obliteration that significantly compromise expansion potential [34]. Importantly, contemporary evidence emphasizes that chronological age represents a weak and unreliable predictor of MPS maturation status and should not serve as the primary criterion for treatment planning decisions. While population-level trends suggest progressive suture ossification during the second and third decades of life, CBCT and microcomputed tomography (µCT) studies have conclusively demonstrated substantial interindividual variability, with documented cases of incomplete suture fusion in patients aged 32–71 years and, conversely, advanced ossification in individuals in their early twenties. Furthermore, CBCT and micro-computed tomography (µCT) studies have demonstrated that palatal bone thickness, cortical bone density, and suture width at specific anatomical landmarks, particularly 18 mm and 21 mm posterior to the incisive foramen, correlate significantly with expansion outcomes, with thinner MPS bone and thicker palatal cortical bone associated with more favorable skeletal responses [26,29].

Beyond suture biology, the influence of craniofacial resistance structures constitutes a paramount factor in determining treatment success or failure. The zygomaticomaxillary buttress and crista zygomaticoalveolaris serve as major points of skeletal resistance, with CBCT-based analyses revealing that the center of rotation for the zygomaticomaxillary buttress during expansion is located near the superior aspect of the frontozygomatic suture, thereby creating apical resistance that contributes to the characteristic pyramidal expansion pattern observed in non-surgical cases [26,28,29]. The pterygopalatine region represents another critical resistance structure; studies have consistently demonstrated that successful disarticulation of the pterygomaxillary suture is significantly associated with greater MPS expansion, with a reported correlation indicating that for each millimeter of pterygoid suture separation, midpalatal expansion increases by 1.46 mm. Conversely, failure to achieve pterygomaxillary disjunction has been identified as a significant predictor of treatment failure, particularly in patients with advanced suture maturation. Additionally, the nasal pillars and anterior maxillary buttresses contribute to expansion resistance in the coronal plane, resulting in greater expansion at the palatal level compared to the nasal cavity (2.99 mm versus 2.24 mm, respectively) [44,45,46]. The high relevance of these resistance structures for treatment failure is evidenced by the observation that all instances of MARPE failure occurred in patients at late stages of suture maturation (stages D and E) and those exhibiting narrower circummaxillary sutures, including the zygomaticomaxillary, pterygomaxillary, and transverse palatine sutures [47].

*Biomechanical integration: force distribution and stress concentration across resistance structures.* Finite element method (FEM) modeling and three-dimensional CBCT-based biomechanical analyses have provided mechanistic insights into how expansion forces are distributed and concentrated across multiple resistance structures during MARPE activation [8,48]. FEM studies have demonstrated that expansion forces applied through miniscrew anchorage are not uniformly distributed but, rather, concentrated at discrete anatomical sites: the zygomaticomaxillary buttress and pterygomaxillary junction serve as primary stress concentration zones, with computed strain magnitudes in these regions exceeding those at the MPS by 2- to 3-fold [48,49,50]. This force concentration pattern explains why the magnitude of skeletal resistance at these sites, determined by bone density, suture width, and structural geometry as assessed on pre-treatment CBCT, becomes a critical determinant of treatment outcome [47]. Specifically, in patients with pronounced zygomaticomaxillary buttress density, thick cortical bone at the crista zygomaticoalveolaris, or narrow pterygomaxillary suture width, the high biomechanical resistance at these sites exceeds the force-generating capacity of the miniscrew anchorage system, resulting in: (1) incomplete or asymmetric MPS separation, (2) disproportionate dentoalveolar tipping as a compensatory mechanism for limited skeletal expansion, (3) increased risk of miniscrew failure due to biomechanical overload at the anchorage sites, or (4) frank treatment failure with absent midpalatal separation despite adequate appliance activation [46,47,48,49]. Conversely, CBCT-based evidence documents that successful MARPE cases are characterized by thinner, less densely ossified pterygoid bones and wider circummaxillary sutures that offer less biomechanical resistance, thereby allowing complete separation of the midpalatal and pterygomaxillary sutures at reasonable force magnitudes [41,47].

### 4.4. Intracranial Effects

From a biomechanical perspective, early finite element method (FEM) models and experimental animal studies suggested that rapid maxillary expansion might induce stress and displacement in cranial base structures, with potential effects on venous and cerebrospinal fluid dynamics. These models demonstrated that forces generated during expansion can be transmitted toward the cranial base and adjacent foramina [8]; however, such simulations and non-human data represent indirect and low-level evidence and do not by themselves establish clinically relevant intracranial consequences in humans [49]. Similarly, isolated case reports have hypothesized a possible association between rapid palatal expansion and symptoms compatible with idiopathic intracranial hypertension, including headache and diplopia; however, these reports are rare, involve small samples, and are subject to substantial confounding [48,49,50,51,52,53,54]. As a result, they should be interpreted as hypothesis-generating signals rather than proof of a causal relationship [48,54].

More recent clinical research using objective intracranial pressure surrogates has not confirmed clinically relevant pressure alterations during MARPE therapy in late adolescents and young adults. In a prospective clinical study, Baser et al. measured optic nerve sheath diameter (ONSD) by ultrasound before and after initial MARPE activation and found no significant changes in ONSD values, suggesting that MARPE does not acutely increase intracranial pressure under standard activation protocols [7]. Likewise, contemporary scoping and systematic reviews that included MARPE and surgically assisted rapid palatal expansion (SARPE) have consistently reported that documented intracranial or neuro-ophthalmologic adverse events are rare, sporadic, and supported only by low-level evidence, particularly when compared with the more frequent and better-characterized dentoalveolar and periodontal complications [15]. When MARPE is contrasted with SARPE in adult patients, the available evidence focuses primarily on skeletal and periodontal outcomes and does not demonstrate a higher incidence of confirmed intracranial complications with MARPE; instead, intracranial or neurovascular events remain largely hypothetical or anecdotal in both modalities [53,54].

From a clinical standpoint, it seems more appropriate to describe these potential effects as rare and largely hypothetical, while still emphasizing the importance of vigilant monitoring for neurological “red flags,” such as persistent or severe headache, visual disturbances (including diplopia or transient visual obscurations), nausea, vomiting, or papilledema [50,51,52], which should prompt immediate medical and neuro-ophthalmologic evaluation. In this context, clinicians are encouraged to (1) obtain a detailed medical and neurological history before MARPE, particularly in patients with pre-existing intracranial or neurovascular disorders; (2) inform patients and caregivers that intracranial complications are considered rare but theoretically possible; and (3) coordinate interdisciplinary management with neurology or ophthalmology when suspicious symptoms arise during or after expansion [50,51,52,53,54].

### 4.5. Study Limitations

The present scoping review also has several important methodological limitations that should be acknowledged explicitly. First, although four major electronic databases were systematically searched, grey literature sources (e.g., conference abstracts, theses, dissertations, and non-indexed reports) were not included, which may have led to omission of relevant but unpublished or non–peer-reviewed data and may have contributed to publication bias toward positive or more “successful” MARPE outcomes. Second, only English-language articles were considered eligible, so potentially informative studies published in other languages were excluded; this language restriction further increases the risk of selection bias and may limit the global generalizability of our findings, particularly given that much of the MARPE literature originates from non-English-speaking countries. Third, although the search strategy was substantially expanded and refined, it still relied on a predefined set of keywords and controlled vocabulary focused on MARPE, treatment failure, and adverse effects; as a result, studies using atypical terminology or reporting MARPE-related complications only as secondary outcomes might have been missed despite being indexed in the searched databases. Finally, several of the included systematic and umbrella reviews synthesized overlapping sets of primary MARPE studies, and those primary studies were also captured directly in our search; while overlapping evidence was carefully identified and treated as non-independent data points, this redundancy—combined with the small number of available primary clinical investigations—may inflate the apparent volume of evidence and further underscores that current conclusions about MARPE efficacy, failure mechanisms, and adverse effects are based on a limited and partially overlapping evidence base rather than on multiple large, independent cohorts.

## 5. Conclusions

The present scoping review maps and synthesizes current evidence on MARPE success rates, treatment failure, and adverse effects in late adolescents and young adults, providing clinically actionable information for everyday orthodontic decision-making. The findings suggest that MARPE can be a viable alternative to surgically assisted maxillary expansion for carefully selected patients, supporting its use particularly in cases with favorable skeletal patterns, adequate bone quality, and realistic expectations regarding possible complications and treatment stability. Clinicians may translate these results into practice by using the reported ranges of success and complication rates to inform case selection, risk communication, and shared decision-making, as well as to guide individualized protocol adjustments (e.g., anchor design, activation schemes, and retention strategies) that aim to minimize adverse outcomes. At the same time, the identified methodological limitations and heterogeneity of the available studies underline the need for well-designed prospective and randomized trials to refine MARPE indications, standardize outcome definitions, and strengthen the evidence base to support clinical implementation.

## Figures and Tables

**Table 1:** Main characteristics, overall risk-of-bias assessment, and main findings from included studies.

Authors, Year	Country	Sample and Characteristics	Intervention	Key Findings and Reported Adverse Effects
**A. Systematic reviews with meta-analysis (2021–2023)**				
Kapetanović et al., 2021 [9]	Netherlands	Adolescents and adults ≥16 years old; all types of MARPE device designs	MARPE with 4 micro-screws in the hard palate	Success rate ~92.5%; significant buccal dental inclination of first molars; various device design specifications
Huang et al., 2022 [25]	China	Patients ≥ 15 years old	MARPE with assessment of success rate, intermolar width, alveolar width, molar inclination, and alveolar crest height	Significant dental inclination and decreased alveolar height (vertical bone loss in the buccal area); evaluation via CBCT, cephalograms, and dental models
Zeng et al., 2023 [18]	China	Adolescent and adult patients ≥ 13.5 years old	Expansion with 4 micro-screws in hard palate; average activation 5.61 ± 1.19 mm; duration 7.6 ± 5.7 weeks	Gingival irritation, increased periodontal probing depth, root resorption/damage, gingival recession, loss of vitality, decreased buccal bone thickness; evaluation via CBCT, cephalograms, dental models, stereophotogrammetry
**B. Umbrella reviews (2022–2025)**				
Ventura et al., 2022 [3]	Brazil	Comprehensive review of 4 systematic reviews; quality assessment revealed low-to-moderate evidence	MARPE technique across included systematic reviews	Significant dental inclination of upper first molars; identified critical quality limitations in underlying systematic reviews
Lázaro-Abdulkarim et al., 2025 [26]	Spain	17 retrospective and prospective studies included, in which participants were mid-to-late adolescents and young adults with transverse maxillary deficiency, aged 13.8–30 years.	All included studies evaluated (MARPE), using CBCT to quantify skeletal and dental effects at the midpalatal suture, nasal cavity, and other craniofacial sutures. Activation protocols varied but typically involved daily screw turns until clinical diastema or target expansion was achieved.	More than half of the studies reported buccal tipping of anchor teeth, reduced buccal bone thickness, and vertical bone loss.
**C. Systematic review without meta-analysis/Scoping reviews (2022–2025)**				
Silva-Sazo et al., 2022 [27]	South Korea	Patients aged 18–25 years; prospective and retrospective studies (1 prospective, 7 retrospective)	Transverse expansion evaluated via multiple modalities	Decreased buccal bone plate thickness of upper first molars; soft-tissue inflammation around micro-screws; buccal alveolar bone inclination; reduced thickness of buccal bone of upper first premolars; apical migration of alveolar crest; transversal asymmetry post-expansion in 51% of cases
Labunet et al., 2024 [28]	Romania	75 publications (2018–2023) involving patients treated with microimplant-based expanders. Most primary studies focused on post-pubertal individuals requiring maxillary expansion. Eighteen articles analyzed adverse effects.	(MARPE) devices—either hybrid tooth–bone-borne or purely bone-borne—anchored with microimplants inserted into the palate. Activation protocols were daily screw turns, and many studies used CBCT to quantify skeletal changes, sutural responses, and dental/periodontal effects.	Buccal tipping of anchor teeth, decreased buccal bone thickness, vestibular bone reduction, increased torque of supporting teeth, periodontal changes, temporary reductions in pulp blood flow, and partial or delayed midpalatal suture repair. Relapse of transverse and nasal expansion was consistently observed.
Sicca et al., 2025 [29]	Italy	26 studies (11 retrospective studies, 12 prospective studies, and 3 randomized clinical trials) were performed in patients over 18 years of age.	The interventions encompassed two primary rapid maxillary expansion techniques: MARPE and SARPE, with variations in surgical approaches and appliance designs.	Dentoalveolar side effects (particularly dental tipping) were predominantly associated with MARPE, while surgical complications were more commonly observed with SARPE. Patient age is a critical determinant for treatment selection.
Da Silva et al., 2025 [30]	Brazil	26 records fulfilled the inclusion criteria: 11 retrospective studies, 12 prospective studies, and 3 randomized clinical trials	Two rapid maxillary expansion appliances: MARPE and SARPE	The majority of MARPE patients exhibited dental tipping and associated dentoalveolar changes. Clinical outcomes are significantly influenced by age and device planning.
**D. Randomized controlled clinical trials (2022–2025)**				
Chun et al., 2022 [17]	South Korea	40 patients; median palatine suture separation evaluation; 3-month treatment duration	Separation of the median palatine suture assessed with skeletal, dentoalveolar, and periodontal measurements via CBCT (axial view)	Greater increase in nasal width at the molar region and greater palatine foramen immediately after expansion and consolidation periods
Parihar et al., 2025 [31]	India	40 adult patients allocated to MARPE (*n* = 20) or SARPE (*n* = 20). No active periodontal disease and no history of prior expanders.	MARPE, twice-daily activation of 0.25 mm per turn until 7–10 mm of expansion, 6 months of retention. Clinical measures included gingival recession (GR), probing depth (PD), and clinical attachment level (CAL), while CBCT-based parameters comprised buccal alveolar bone thickness and height and volumetric root resorption.	MARPE offers a biologically gentler expansion modality, providing similar skeletal gain with reduced periodontal morbidity—less gingival recession and better preservation of buccal alveolar bone—compared with SARPE.
**E. Pilot cohort study (2018)**				
Ngan et al., 2018 [32]	USA	8 patients; cervical vertebral maturation stage (CVM) ≥ 4; mean age 21.9 years	Before-and-after expansion measurements; assessment of molars and premolars	Significant buccal inclination of upper first molars and first premolars; buccal bone thickness recovery observed at 3, 6, and 24 months post-treatment
**F. Cross-sectional studies (2022)**				
Baser et al., 2022 [7]	Turkey	15 patients; optic nerve sheath diameter (ONSD) measurement via ultrasound	Ultrasound measurement immediately before first activation and at 1 min and 10 min after first activation	No adverse effects reported; no alterations in intracranial pressure during active MARPE therapy in late adolescents
**G. Retrospective studies (2023–205)**				
Chen et al., 2023 [33]	China	22 patients; assessment at 1.5 years post-treatment	Evaluation of interdental papilla height between maxillary central incisors via frontal intraoral photographs using Jemt classification	Significant reduction in Jemt score post-MARPE (18% prevalence); interincisal papilla recession resulting in “black triangle” esthetic concern; difficulty in periodontal regeneration
Rajalakshmi et al., 2025 [34]	India	24 consecutive MARPE patients (mean age of 17.4 ± 1.3 yrs), presenting transverse maxillary deficiency and managed with either hybrid or bone-borne miniscrew-supported expanders.	Two-screw hybrid MARPE or a four-screw MSE-type skeletal expander. All patients underwent activation protocols aiming at “full expansion.” Complication recording included clinical and radiographic evaluation.	Miniscrew fracture, appliance arm breakage, loss of acrylic or connecting components, and screw displacement. Furthermore, tongue irritation/swelling and peri-implant mucositis due to plaque accumulation were assessed.

## Data Availability

The original contributions presented in this study are included in the article and Supplementary Material. Further inquiries can be directed to the corresponding author.

## Supplementary Materials

The following supporting information can be downloaded at: https://www.mdpi.com/article/10.3390/dj14050261/s1, Table S1: Comprehensive search strategies used for each database.
