# Supplementary material for: Clinical Outcomes, Success/Failure Patterns, and Complications of Microscrew-Assisted Rapid Palatal Expansion in Post-Pubertal Transverse Maxillary Deficiency: A Scoping Review
Source: Dent J (Basel). 2026 May 1;14(5):261. doi: 10.3390/dj14050261 (PMC13206503; doi:10.3390/dj14050261)
Supplement: Supplementary file 1 [file dentistry-14-00261-s001.zip › dentistry-4051009-supplementary.pdf]

Supplementary Table S1. Comprehensive search strategies used for each database.

| Database | Search date | Search Fields / Operators | Controlled vocabulary terms (MeSH / Emtree) | Free-text keywords | Filters / limits |
|---|---|---|---|---|---|
| **PubMed (via MEDLINE)** | [Insert date] | Title/Abstract, MeSH | "Palatal Expansion Technique"[MeSH]; "Orthodontic Anchorage Procedures"[MeSH]; "Treatment Failure"[MeSH]; "Drug-Related Side Effects and Adverse Reactions"[MeSH]; "Risk Factors"[MeSH]; "Adolescent"[MeSH]; "Young Adult"[MeSH] | MARPE OR "Mini-implant assisted rapid palatal expansion" OR "Micro-implant assisted expansion" OR "maxillary skeletal expander" OR "bone-borne expander" OR "orthodontic anchorage" AND "failure" OR "adverse effect*" OR "complication*" | Language: English; Year: 2015–2025; Study type: Articles and Reviews |
| **EMBASE (via Ovid)** | [Insert date] | ti,ab,kw; Emtree controlled vocabulary; adjacency (adj2, adj3) | exp palate expansion/; exp orthodontic anchorage/; exp treatment failure/; exp adverse drug reaction/; exp complication/; exp risk factor/; exp periodontal disease/; exp tooth resorption/ | MARPE OR "mini-implant assisted rapid palatal expansion" OR "maxillary skeletal expander" OR "bone-borne expander" OR "orthodontic anchorage" AND "failure" OR "side effect*" OR "complication*" | Language: English; Year: 2015–2025; Document type: Article, Review |
| **Scopus** | [Insert date] | TITLE-ABS-KEY | — | ("MARPE" OR "mini-implant assisted rapid palatal expansion" OR "bone-borne expander" OR "maxillary skeletal expander") | Language: English; Years: 2015–2025; Document type: Article, Review; |

| | | | | | |
|---|---|---|---|---|---|
| | | | | AND (“failure” OR “adverse effect*” OR “complication*”) AND (“adolescent” OR “young adult”) | Excluded: case reports, letters, editorials, notes |
| **Cochrane Library** | [Insert date] | ti,ab,kw; MeSH (exploded) | “Palatal Expansion Technique”[MeSH]; “Orthodontic Anchorage Procedures”[MeSH]; “Treatment Failure”[MeSH]; “Adverse Effects”[MeSH]; “Risk Factors”[MeSH] | MARPE OR “mini-implant assisted rapid palatal expansion” OR “skeletal expander” OR “orthodontic anchorage” AND “failure” OR “complication*” | Publication type: Trials, Reviews; Date: 2015–2025; Language: English |
